# Evolutions and Managements of Soil Microbial Community Structure Drove by Continuous Cropping

**DOI:** 10.3389/fmicb.2022.839494

**Published:** 2022-02-28

**Authors:** Yudong Chen, Jianfeng Du, Yang Li, Heng Tang, Ziyi Yin, Long Yang, Xinhua Ding

**Affiliations:** State Key Laboratory of Crop Biology, Shandong Provincial Key Laboratory for Biology of Vegetable Diseases and Insect Pests, College of Plant Protection, Shandong Agricultural University, Tai’an, China

**Keywords:** soil ecosystem, continuous cropping obstacles, microbial community structure, management measures, evolutions

## Abstract

Continuous cropping obstacles have increasingly become an important phenomenon affecting crop yield and quality. Its harm includes the deterioration of soil basic physical and chemical properties, changes of soil microbial community structure, accumulation of autotoxins, weakness of plant growth, and aggravation of diseases and pests. In this review, the evolutionary trend of soil microbial structure driven by continuous cropping was generalized, while drivers of these changes summed up as destruction of soil microbial living environment and competition within the community. We introduced a microorganism proliferation and working model with three basics and a vector, and four corresponding effective measures to reshape the structure were comprehensively expounded. According to the model, we also put forward three optimization strategies of the existing measures. In which, synthetic microbiology provides a new solution for improving soil community structure. Meanwhile, to ensure the survival and reproduction of soil microorganisms, it is necessary to consider their living space and carbon sources in soil fully. This review provided a comprehensive perspective for understanding the evolutionary trend of the soil microbial community under continuous cropping conditions and a summary of reshaping measures and their optimization direction.

## Introduction

Although China has more than 5.28 million square kilometers of agricultural land, its population has already reached 1.41 billion, needing more safe food. Therefore, scientists and farmers are seeking safety, quality, and high yields while exploiting the land’s capacity to produce more food (Wang et al., 2018a). However, limited by various geographical factors, farmers seldom adopt a rotation system, but rather more continuous planting. Finally, continuous cropping obstacles have formed, bringing about various ecological and environmental problems.

**Figure 1:**
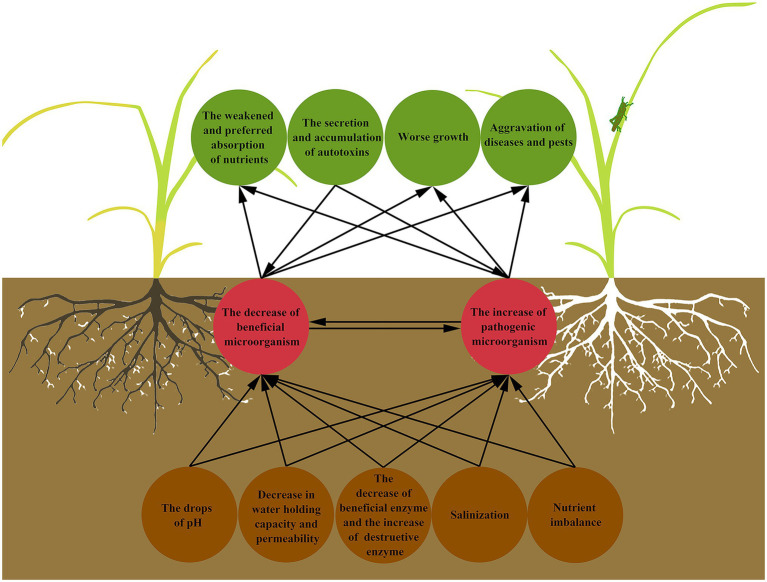
Interactions between the soil microbial community and the manifestation of continuous cropping obstacles. Continuous cropping results in deterioration of soil physical and chemical properties, which further affects the survival, proliferation, and working of soil microorganisms, and destroys soil microbial community structure. When it comes to plants, it is the abnormal growth state, the aggravation of diseases and insect pests, and the weakened and preferred absorption of nutrients. At the same time, the autotoxins secreted by plants affect the soil microorganisms in turn.

Continuous cropping obstacles refer to the phenomenon that the same crop or its related species are continuously planted on the same plot, and even under normal management conditions, the yield and quality of products are still reduced, and the diseases and insect pests become serious (Yan et al., 2012; Xi et al., 2019; Chen et al., 2020; Wang et al., 2020c). Continuous cropping obstacles are also known as “avoid land,” “replanting disease,” “hate land problem,” and “repeat crop”(Zhou and Wu, 2012; Wang et al., 2015). Continuous cropping can lead to the decrease of soil available nutrients contents (Li et al., 2019), the imbalance of nutrient elements proportion (Yu et al., 2017), the decline of soil enzyme activity, the deterioration of physical and chemical properties (Du et al., 2017), the changes of microbial population, and the aggravation of diseases and pests (Zhang et al., 2017; Zeng et al., 2020; Figure 1). The decline of crop yield and quality is the final result and manifestation of the above hazards and also indicates the decrease of farmers’ income (Zhang, 2015; Chen et al., 2018c), which makes the alleviation or removal of continuous cropping obstacles become a major problem in the process of planting, and also a most challenging problem to solve.

Microorganisms are another kind of material and energy carrier besides plants and animals in the soil (Maier et al., 2018; Macik et al., 2020). Their existence can efficiently transform all kinds of energy (Victoria et al., 2013; Venkatesh and Pradeep, 2016) and effectively impact the soil structure and quality (Jangir et al., 2019; De Corato, 2020). Microorganisms also play an important role in crop growth and health (Schippers et al., 1987; Judith and Donald, 2020) and become an essential indicator to measure soil health (Syrie et al., 2017; Johannes et al., 2020). Long-term continuous cropping of the same plant or the same family plants causes changes in the quantity, diversity, and richness of soil microorganisms (Li and Liu, 2019; Liu et al., 2020a,c), and the occurrence of continuous cropping obstacles is closely related to the imbalance of soil microbial community structure (Zhang et al., 2019; Liu et al., 2021b).

In this review, the evolutionary trend of soil microbial community structure driven by continuous cropping and drivers of the evolution were generalized. Meanwhile, we raise a microorganism proliferation and working model and management measures to overcome the continuous cropping obstacles. Finally, improvements to existing measures were also proposed. We hope this review provides a comprehensive landscape for comprehending the soil microbial community under continuous cropping conditions and supports the final mitigation of continuous cropping obstacles.

## Evolutions of the Assembly of Soil Microbial Community Drove by Continuous Cropping

Soil microbial community structure affects plant health and can also be used as an indicator of soil health (Zhou and Wu, 2012; Dong et al., 2016). High-throughput sequencing analysis showed that long-term continuous cropping could reduce soil bacterial biomass and increase fungal biomass (Dong et al., 2016; Liu et al., 2016, 2020a), while longer-term continuous cropping could increase the bacterial diversity to suppress soil-borne diseases by forming bacteriostatic soil (Shen et al., 2018; Zhang et al., 2020). Studies have shown that the higher the ratio of bacterial to fungal in soil, the better condition of the soil ecosystem, the more stable the structure of bacteria, and the stronger the resistance of soil to disease (Liu et al., 2015a,b). After continuous cropping, the dominant microorganism in soil changed from bacteria to fungi, while the number and diversity of fungi was negatively correlated with the soil health status (Han et al., 2010). Among the proliferative fungi in the soil, some fungi can directly kill plant cells or produce metabolic toxins (Zhu et al., 2014; Manici et al., 2017). These fungi also directly affect the health of the whole plant by destroying root growth and physiological activities (Yim et al., 2013; Emmett et al., 2014). Continuous cropping of common buckwheat significantly increased the number of rhizosphere fungi, decreased soil nutrient content, enzyme activity, feedback for agronomic traits, and root index decreased significantly (Wang et al., 2020c). After planting apple seedlings in continuous cropping fields, the root system showed necrosis of epidermal cells, root tip rot, lateral root development retarded, and functional root hairs decreased due to the action of fungi in soil (Mazzola and Manici, 2012; Yim et al., 2013; Emmett et al., 2014; Weiβ et al., 2017).

With the increase in continuous cropping years, the diversity and richness of the rhizosphere microbial community changed greatly, the number of functional strains (Wang et al., 2018b), such as aerobic bacteria and nitrogen-fixing bacteria, decreased significantly, and the diversity index of soil fungi and bacteria declined, which destroy the balance of original soil microbial community structure and effected plant growth (Larkin, 2008; Mikkel et al., 2015). Some bacteria, which belong to plant growth promoting rhizobacteria (PGPR), can secrete antibiotics to inhibit pathogenic microorganisms. Still, continuous cropping reduces bacteria-secreted antibiotics (Zhang et al., 2020) and the inhibition of pathogenic bacteria (Tan et al., 2017) and then causes an increase in plant diseases incidence. Meanwhile, the secondary metabolites secreted by harmful microorganisms recruit microorganisms that are beneficial to themselves, further damaging the soil microecology, evolving in a direction conducive to the survival of those, and aggravating the obstacles of continuous cropping (Chen et al., 2018a; Pascale et al., 2019; Bakker et al., 2020).

## Drivers of Changes in Soil Microbial Community Structure Under Continuous Cropping

**Figure 2:**
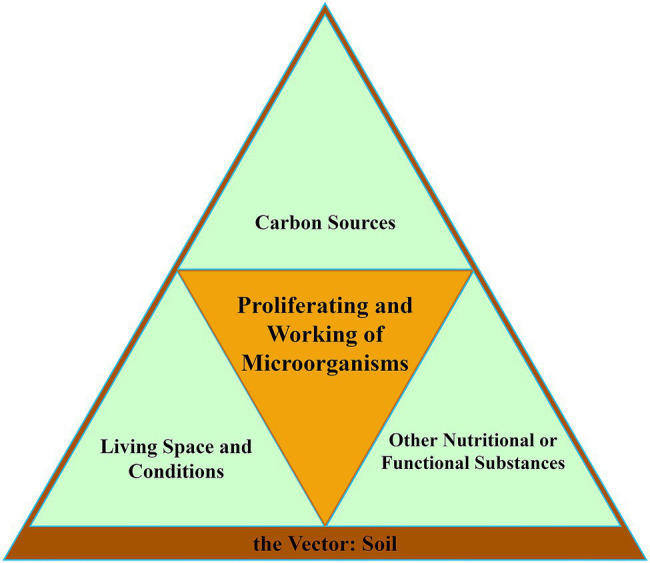
Three basics and a vector for proliferating and working of soil microorganisms.

The ability of soil microorganisms to survive, proliferate, and work in an environment requires certain specific foundations. To sum up, there are three basics and one vector. We constructed them into a microorganism proliferation and working model (Figure 2). The first basic is carbon sources, the essential nutrient for microbial life activities. The second is the living environment, including living space and living conditions. The last one is other nutritional and functional substances, which are other substances necessary for the survival and working of microorganisms. These three basics exist in a common vector, the soil. The deterioration of any one of the three basics will lead to the retardation of proliferation or working of microorganisms. According to this model, we analyzed the drivers of microbial evolution trends under continuous cropping.

### Changes in Soil Physical and Chemical Properties Rebuild the Living Environment of Microorganisms

The changes of soil physical and chemical properties directly or indirectly lead to the formation of continuous cropping obstacles and rebuild the living environment of microorganisms. Studies have shown that continuous cropping and excessive use of chemical fertilizers and pesticides lead to a decline in soil pH (Serpil, 2012; Hvězdová et al., 2018), which accelerates the colonization of pathogenic microorganisms and aggravates plant diseases (Joseph et al., 2018). At the same time, the absorption of nutrient elements by single crops is biased, resulting in the imbalance of soil nutrients, which accelerates the evolution of microbial communities (Pan et al., 2014; Wang et al., 2017). Excessive use of chemical fertilizers and pesticides also leads to salinization and hardening (Liu et al., 2015b; Shen et al., 2016), which will further increase soil osmotic potential, decrease buffer capacity, reduce aggregate structure, and decline the water holding capacity and permeability (Serpil, 2012; Hvězdová et al., 2018). Meanwhile, soil catalase and other harmful enzymes accumulated with the increase of continuous cropping years, further destroying the living conditions of soil microorganisms (He et al., 2008; Huang et al., 2012). These changes will eventually lead to variations in the living environment of microorganisms, and some particular microorganisms will be recruited or selected to adapt to the new rigorous environment and gradually change the soil microbial community.

### Autotoxins Lead to a Decrease in Probiotics

Although plant root exudates varied in types and functions, autotoxins are another factor leading to the change of soil microbial structure in continuous cropping obstacles. Many plants can release some substances through aboveground volatilization, leaf leaching, eluviation, root secreting, and plant stubble decaying, which can inhibit the growth of this season crop or the next season crop of the same species or the same family of plants (Rial et al., 2014; Hisashi et al., 2017). This phenomenon is called autotoxicity or allelopathy inhibition (Friedman, 2017). These released substances are mainly secondary metabolites, known as autotoxins, primarily phenolic acids (Zhang et al., 2007; Rial et al., 2014). The autotoxins in tobacco that have been detected and verified included benzoic acid, p-hydroxybenzoic acid, vanillic acid, vanillina, etc., among which benzoic acid has the most significant allelopathy effect (Wu, 2010). With the addition of continuous planting years, the accumulation volume of autotoxins increases with soil acidification (Wang et al., 2008; Wu et al., 2015). The inhibition effect on probiotics, related to element circulation and soil texture improvement, becomes more and more intense (Kumar et al., 2017; Furtak and Gajda, 2018). At the same time, the accumulation of autotoxins provides carbon sources for pathogenic microorganisms, and the growth-promoting effects on pathogens begin to appear (Zhao et al., 2015; Chen et al., 2018b; Jia et al., 2018). Finally, pathogens occupy more favorable ecological niches, disrupting the balance of small underground ecosystems.

### Competition Between Pathogenic Microorganisms and Probiotics

The competition between pathogenic microorganisms and probiotics is another reason for the change of soil microbial community structure (Griffin et al., 2004; Stéphane et al., 2013). Specifically, it is the competition between them for living space and resources (Amin et al., 2020; Gu et al., 2020). Under the ground, space and resources are common to both. The one that can reproduce quickly will take up more space and resources and occupy a reasonable ecological niche, especially for scarce resources, such as siderophores (Gu et al., 2020). Accordingly, when the quantity of probiotics is artificially added to counter pathogens, the living environment should be improved at the same time, and the reproduction of probiotics will be accelerated (Jin, 2010). However, there are still some factors in the soil that affect their competition, such as predators and plants. Predation behaviors reduce the survival of one of them, and the competition between them becomes more fierce (Rasit et al., 2021). To ensure the balance between them, plants recruit probiotics and fight against pathogens by secreting their products, intensifying their competition and even breaking the balance (Sassone-Corsi and Raffatellu, 2015; Liu et al., 2021a). With the increase of continuous cropping years, all kinds of resources and space in the soil are gradually occupied by pathogens, probiotics lose their ecological niche, and crop growth status becomes worse and worse (Pervaiz et al., 2020). However, if farmers continue to choose continuous cropping, likely, probiotics will gradually accumulate due to the recruitment of plants, and the living space and resources of pathogens will be squeezed continuously, eventually forming bacteriostatic soil.

## Improvement Measures of Soil Microbial Community Structure Under Continuous Cropping

**Figure 3:**
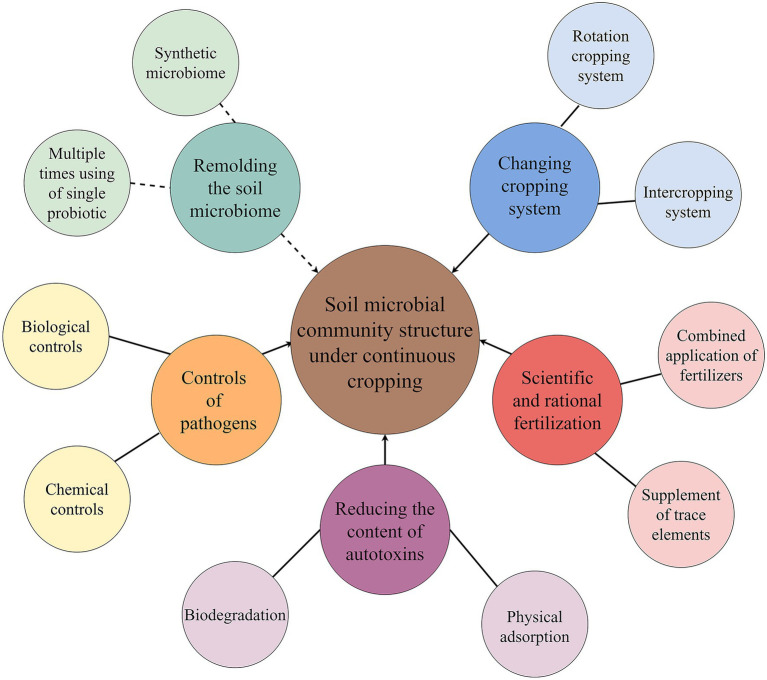
Improvement measures of soil microbial community structure under continuous cropping. The solid line indicates the measures currently used in agricultural production, and the dotted line indicates the measure that will be involved in the mitigation of continuous cropping obstacles in the future.

In recent years, the mitigation of continuous cropping obstacles has become a hot issue to be solved urgently in production (Liu et al., 2020a,c; Zeng et al., 2020; Ding et al., 2021). One of these methods that can mitigate continuous cropping obstacles is improving or recombining microbial community structure underground (Larkin, 2008; Han et al., 2010; Liu et al., 2015a, 2021a; Chen et al., 2018c; Zhang et al., 2020). Special cultivation control measures can solve some problems of soil community structure caused by continuous cropping, but most of them have no feasible reduction techniques. At present, there are several ways or measures to improve the microbial community structure of continuous cropping soil (Figure 3).

### Changing the Current Cropping System

The soil ecological damage caused by planting the same or the same family of plants for many years could be changed by altering the existing planting system to carry out reasonable rotation cropping or intercropping, and the change of planting system can alter the structure of microflora (Larkin, 2008; Du et al., 2017; Gao et al., 2017; Zeng et al., 2020). Changes or increases in crop species lead to changes in root secretions in soil (Galazka et al., 2017; Li et al., 2020). Thus, microorganisms that use the new secretions as a carbon source are increased or recruited, rebuilding the microbial community. Three kinds of crops, sweet potato, peanut, and wheat, were cropped rotationally, and the results showed that the number of culturable bacteria in sweet potato, peanut rotation cropping field was increased compared with that in continuous cropping soil, the quantities of fungi and actinomycetes were decreased, and the contents of nutrient elements in the soil were changed (Fan, 2019). At the same time, the quantities of culturable bacteria and fungi in the wheat field showed an increased tendency, but a decreased trend of actinomycetes. However, the sweet potato–tobacco intercropping increased the dominant microflora at the phylum level and changed the microflora structure. The soil microecological system in the root zone became more and more benign. The intercropping between peanut and tobacco can also optimize the soil microecological structure in the peanut continuous cropping field (Gao et al., 2019). It is worth noting that regardless of whether crop rotation or intercropping cropping patterns are used, the choice of crops is critical, especially not to choose crops with co-morbidities (Chongtham et al., 2017; Ouda et al., 2018). To sum up, selecting suitable crops for rotation cropping or intercropping is very important for mitigating soil community structure and improving plant growth and yield quality.

### Scientific and Rational Fertilization

Scientific and reasonable fertilization strategy is the necessary measure to maintain the balance of soil nutrient elements and the living space of microorganisms, because continuous cropping amplifies the preference of the assimilation of elements, changes the physical and chemical properties of the soil, and destroys the living space and nutrients of soil microorganisms (Serpil, 2012; Liu et al., 2015a; Chen et al., 2018c; Li et al., 2019; Macik et al., 2020). A rational choice of fertilization strategy based on soil conditions is the only option to stabilize soil physicochemical properties and maintain soil microbial nutrients (Zhao et al., 2014). But in production, farmers generally only pay attention to the application of fertilizers rich in macroelements, such as nitrogen, phosphorus and potassium fertilizer, ignoring the trace elements fertilizer and organic fertilizer (Michalojc and Buczkowska, 2009; Shen et al., 2016; Kicinska and Wikar, 2021). This disrupts the buffering capacity and ionic balance of the soil, lowers the pH value, reduces the effectiveness of certain nutrients, and leads to the lack of essential plant nutrients, such as Ca, Mg, B, Mo (Zhenli et al., 2005; Amir et al., 2019; Macik et al., 2020). Ultimately, a variety of physiological and soil-borne diseases to crop plants happened. The reasonable addition of microelement fertilizers to the soil can alleviate nutrient deficiencies caused by preferential absorption of crops, help maintain stable soil physical and chemical properties and microbial communities, and enhance plant health and immune resistance.

Farmers also can use the competitive relationship between probiotics and pathogens, through the application of bacterial fertilizer, to increase the quantity of probiotics, but have better to create an appropriate living environment for the survival of probiotics at the same time, to promote their rapid reproduction (Zhang et al., 2012; Liu et al., 2015a; Chen et al., 2018c; Li et al., 2019; Sadikshya et al., 2020). The combined application of organic fertilizer, microbial fertilizer, and chemical fertilizer can not only increase the contents of various nutrient elements and soil organic matter and improve the physical and chemical properties of soil to a certain extent, but also optimize the soil microbial population and structure, and increase the biomass of probiotics in soil (Miransari, 2013; Dubey et al., 2020, 2021). At the same time, combined application of fertilizers can balance the chemical composition of plants, increase the total content of inclusions and improve the quality of harvests (Dubey et al., 2019, 2020). In conclusion, the compound application of all kinds of fertilizer has obvious effects on alleviating continuous cropping obstacles. In addition, specific plans for the application ratio of organic fertilizer, microbial fertilizer, and chemical fertilizer should be made according to local soil characteristics.

### Adsorption and Degradation of Autotoxins

Reasonable planting system and fertilizer collocation are the first choice to degrade autotoxins, which is simple and labor-saving. And reducing the content of autotoxins in the root zone is another method to improve microbial community structure (Zhang et al., 2016; He et al., 2021). Physical adsorption and biodegradation can also be used to reduce autotoxicity and improve soil microbial community structure (Mao et al., 2010; Wu et al., 2015; Xie and Dai, 2015; Xia et al., 2019). The quickest way to overcome the deterioration of the microbial community caused by the accumulation of autotoxins is by using adsorbents to remove autotoxins from the root zone (Asao et al., 2004; Palansooriya et al., 2020). Biochar has been primarily used in agriculture in recent years, which is a solid product produced by pyrolysis of organic biomass at high temperatures in an anoxic environment (Elmer and Pignatello, 2011; Xia et al., 2019; Sadikshya et al., 2020). Because of its large porosity and specific surface area, biochar can provide space for microorganisms to survive and proliferate while adsorbing harmful substances in the soil, so it is widely used for soil improvement (Fang et al., 2020; Wang et al., 2020a). The application of biochar reduced the content of autotoxins in the field, weakened the effects of autotoxins on plants’ growth, and increased the biomass, growth rate, and sporulation of probiotics (Wang et al., 2020b; Ma et al., 2021).

The degradation of autotoxins depends mainly on soil microorganisms, also known as autotoxins biodegradation (Mao et al., 2010; Xie and Dai, 2015; Wang et al., 2021). The bacteria isolated from soil had a certain ability to decompose the autotoxins secreted by plant roots, especially when fed back to the soil from which the bacteria isolated (Shen et al., 2020; Wang et al., 2021). Therefore, using beneficial microorganisms can also solve or alleviate autotoxicity. Meanwhile, exogenous microbial inoculation can also promote the breeding of many beneficial microbial communities in the rhizosphere of crops, inhibit the growth of harmful microorganisms and reduce the accumulation of pathogenic bacteria (Xie et al., 2020). However, biological control also has problems, such as high cost and unclear impact on the other organism.

### Controlling the Biomass of Pathogens

The controls of pathogenic microorganisms in continuous cropping soil are the most direct improvement method for soil microorganisms. In recent years, chemical control and biological control have mainly been used in production (Liu et al., 2018; Goring, 2019; Dilzahan et al., 2020; Bindumadhavi and Gopi, 2021). The pathogens that cause continuous cropping diseases are mainly hidden in soil or crop residues over the winter. Thus, it can be reduced by fumigation treatment with chemical agents (Bindumadhavi and Gopi, 2021). Taking soil-borne diseases controls in the tobacco field as an example, researchers found that under the condition of inoculation and common field, the control effect of chloropicrin fumigation on the black shin and root knot nematode disease reached 68.00% ~ 84.29, 80.66% ~ 92.49, 75.16% ~ 88.15, and 53.60% ~ 65.70%, respectively (Wang et al., 2010). Three extracts from medicinal crops were used to control the tobacco black shank, and the results showed whether used alone or in combination, the antifungal effect was obvious (He et al., 2017). In actual production, because of the broad-spectrum of chemical controls, the controls of soil-borne diseases are more in favor of biological control. Biological controls can reduce the number of pathogenic organisms through probiotics (Liu et al., 2018) or promote soil microorganisms gathering around plant roots to form a sticky layer, which effectively prevents the spread of pathogens later, and reduces the invasion of those to plant roots (Ren et al., 2015; Wu et al., 2019). Many types of bacteria are used for biological control, such as *Bacillus* sp. and *Pseudomonas sp.* for tobacco cultivation (Ma et al., 2017). For example, three strains of endophytic bacteria 001, 009 and 011, isolated from tobacco stems, had a good control effect on tobacco bacterial wilt. Among them, strain 001 was *Bacillus subtilis*, and strain 009 and strain 011 were *Bacillus brevis*. The average control effect of the three strains on tobacco bacterial wilt was 82.5, 100 and 84.5%, respectively (Yin et al., 2004). A new organic fertilizer with *Pseudomonas aeruginosa* NXHG29 could more effectively decrease the disease incidence of tobacco bacterial wilt and tobacco black shank (Ma et al., 2018). Biocontrol bacteria XE01 and X23 could reduce the incidence and severity of tobacco bacterial wilt (Zhou et al., 2016), and endophytic bacteria LSN02 and LLGJ04 were used to control soil-borne diseases of tobacco with results showed that root irrigation by endophytic bacteria was effective and had a significant effect on the control of black shack (Liu et al., 2019). Arbuscular mycorrhizal fungi (AMF) are also widely used probiotics. After applying *Panax quinquefolius* in a continuous cropping field, soil microbial community structure was improved, with functional strains increased and pathogenic microorganisms decreased (Liu et al., 2020b). The plate confrontation test showed that AMF could inhibit the growth of *Verticillium dahliae* and improve the resistance to *Verticillium* Wilt in cotton fields (Zhang et al., 2018).

## Prospects for the Reconstitution of Soil Microbial Structure

The healthy growth of crops is inseparable from a healthy soil ecology. In which, the healthy soil microbial community structure is fundamental. The structure should generally be a bacteria-dominated community without microorganisms harmful to crop growth. Reasonable microbial population, high diversity and stable structure are also essential. Besides, sufficient and various nutrition, adequate living space and suitable living conditions are the premise of a stable community structure. The above measures are the targeted measures to reshape the soil microbial community structure commonly used in agricultural production at present.

However, the basis for the effectiveness of microorganisms is their ability to survive and proliferate in the soil, which requires carbon sources, living space and conditions, and other nutrients and functional substances (Figure 2). To fundamentally solve this problem, it is necessary to use more in-depth means or measures, such as synthetic microbiology, to reshape soil microbial community structure (Du et al., 2020). Researchers have changed from using a single microbe for restoration to using a complex microbiome for reconstruction, which is the source of the formation of synthetic microbiology. With sequencing technology development, more and more high-throughput synthetic microbial communities will be applied to the functional study of soil microbial community structure. Results showed that a simplified synthetic community composed of three high-abundance bacteria and one low-abundance bacteria could control the root rot of *Astragalus mongolicus* (Li et al., 2021). It is inferred that synthetic microbial communities based on major components, functions, and phylogenetic relationships will be generated soon to improve the microbial community structure under continuous cropping obstacles (Du et al., 2021).

The existence of organisms cannot be separated from a reasonable and comfortable living space, so are the microorganisms in the soil. Appropriate living space is needed for both the probiotics in continuous cropping soil and the probiotics added artificially. Most common microbial fertilizers in the market do not fully consider the amount of biomass of active microorganisms after the application, leading to a large amount of bacterial fertilizer used for each season, but the effect is down to expectations. To ensure the survival of exogenous probiotics, their living space must be fully considered. In the present application research, biochar provides reasonable space for probiotics to live, which is determined by the diversity of biochar space structure and the structural properties of the existence of voids. Subsequent production of biological fertilizer can use a combination of biochar and probiotics to increase the survival medium of microorganisms (Elmer and Pignatello, 2011; Fang et al., 2020; Sadikshya et al., 2020; Wang et al., 2020a,b; Ma et al., 2021).

The survival of microorganisms also requires carbon sources. In the reshaping process of soil microbial community structure, adding carbon sources available to probiotics but unavailable to pathogens is an effective measure to control the direction of reshaping. Although continuous cropping recruited probiotics to resist pathogens, the use of specific carbon sources helped speed up the process of reshaping and control the reshaping direction (Yang et al., 2019; Du et al., 2021). In the production and application of agricultural microbial preparations, carbon sources should be added according to the metabolic characteristics of probiotics to improve the effectiveness of the preparations.

## Concluding Remarks

Continuous cropping leads to a change in soil from bacterial type to fungal and reduces the probiotics biomass in soil. The drivers of these changes are the deterioration of soil physicochemical properties, the accumulation of autotoxins, the reduction of beneficial bacteria, and the multiplication of pathogens, further leading to the destruction of the microbial living environment and the increase of competition within the community. Nevertheless, more rigorous experiments should be designed to verify the specific reasons for the changes in soil microbial community structure induced by continuous cropping. We also put forward a microorganism proliferation and working model with soil as a vector. In view of the above drivers and the model, methods adopted in the production include changing the planting system, scientific and rational fertilization, adsorption and degradation of autotoxins, controls of pathogens colonization, and increases in probiotic biomass. Recently, scientists have begun to use complex microbial products, especially synthetic microbiology products, or in combination with other measures, to increase probiotics’ survival, improve soil community structure and relieve continuous cropping obstacles. This is also a direction of application in the future for a period of time. In conclusion, the mitigation measures for the deterioration of microbial community structure driven by continuous cropping can be concluded as the timely reconstruction of soil microbial community structure after continuous cropping, while maintaining the living space and conditions suitable.

## Author Contributions

YC: conceptualization, visualization, writing—original draft preparation, and writing—review and editing. JD and HT: investigation and writing—review and editing. YL: investigation, supervision, and writing—review and editing. ZY: supervision and writing—review and editing. LY and XD: conceptualization, validation, and funding acquisition. All authors contributed to the article and approved the submitted version.

## Funding

This work is supported by the Shandong Province Key Research and Development Plan (2019JZZY020608, 2020CXGC010803, and 2019GNC106152), National Natural Science Foundation of China (32072500 and 31872925), Natural Science Outstanding Youth Fund of Shandong Province (JQ201807), Science and Technology Support Plan for Youth Innovation of Colleges and Universities of Shandong Province (2019KJF023), and the Foundation of Shandong Province Modern Agricultural Technology System Innovation Team (SDAIT-25-01).

## Conflict of Interest

The authors declare that the research was conducted in the absence of any commercial or financial relationships that could be construed as a potential conflict of interest.

## Publisher’s Note

All claims expressed in this article are solely those of the authors and do not necessarily represent those of their affiliated organizations, or those of the publisher, the editors and the reviewers. Any product that may be evaluated in this article, or claim that may be made by its manufacturer, is not guaranteed or endorsed by the publisher.

## References

[ref1] Amin M. K. Khadijeh A. Ismail E. Mariana B. S. Rodrigo B. A. O. Hedayat H.. (2020). Interactions between probiotics and pathogenic microorganisms in hosts and foods: a review. Trends Food Sci. Technol. 95, 205–218. doi: 10.1016/j.tifs.2019.11.022

[ref2] Amir R. Sina S. M. Mahdi G. Saeid H. Kosar G. Jelena P.-D. (2019). The influence of chemical, organic and biological fertilizers on agrobiological and antioxidant properties of Syrian Cephalaria (*Cephalaria Syriaca* L.). Agriculture 9:122. doi: 10.3390/agriculture9060122

[ref3] Asao T. Kitazawa H. Tomita K. Suyama K. Yamamoto H. Hosoki T.. (2004). Mitigation of cucumber autotoxicity in hydroponic culture using microbial strain. Sci. Hortic. 99, 207–214. doi: 10.1016/S0304-4238(03)00098-0

[ref4] Bakker P. Berendsen R. L. Van Pelt J. A. Vismans G. Yu K. Li E.. (2020). The soil-borne identity and microbiome-assisted agriculture: looking back to the future. Mol. Plant 13, 1394–1401. doi: 10.1016/j.molp.2020.09.017, PMID: 32979564

[ref5] Bindumadhavi G. Gopi R. (2021). “Exploitation of biofumigation and biocontrol agents for the management of soil-borne diseases,” in Innovative Approaches in Diagnosis and Management of Crop Diseases: Volume 2: Field and Horticultural Crops. ed. Singh R. K., Gopala (CPC Press), 48.

[ref6] Chen S. Qi G. Luo T. Zhang H. Jiang Q. Wang R.. (2018a). Continuous-cropping tobacco caused variance of chemical properties and structure of bacterial network in soils. Land Degrad. Dev. 29, 4106–4120. doi: 10.1002/ldr.3167

[ref7] Chen W. Teng Y. Li Z. Liu W. Ren W. Luo Y.. (2018c). Mechanisms by which organic fertilizer and effective microbes mitigate peanut continuous cropping yield constraints in a red soil of south China. Appl. Soil Ecol. 128, 23–34. doi: 10.1016/j.apsoil.2018.03.018

[ref8] Chen P. Wang Y.-Z. Liu Q.-Z. Zhang Y.-T. Li X.-Y. Li H.-Q.. (2020). Phase changes of continuous cropping obstacles in strawberry (Fragaria × ananassa Duch.) production. Appl. Soil Ecol. 155:103626. doi: 10.1016/j.apsoil.2020.103626

[ref9] Chen S. Yu H. Zhou X. Wu F. (2018b). Cucumber (*Cucumis sativus* L.) seedling rhizosphere *Trichoderma* and *Fusarium* spp. communities altered by Vanillic acid. Front. Microbiol. 9:2195. doi: 10.3389/fmicb.2018.02195, PMID: 30283420 PMC6157394

[ref10] Chongtham I. R. Bergkvist G. Watson C. A. Sandstrom E. Bengtsson J. Oborn I. (2017). Factors influencing crop rotation strategies on organic farms with different time periods since conversion to organic production. Biol. Agric. Hort. 33, 14–27. doi: 10.1080/01448765.2016.1174884

[ref11] De Corato U. (2020). Towards new soil management strategies for improving soil quality and ecosystem services in sustainable agriculture: editorial overview. Sustainability 12:9398. doi: 10.3390/su12229398

[ref12] Dilzahan H. A. Yoshida M. Umeda M. Calubaquib M. A. Organo N. D. Cruz A. F. (2020). Biocontrol of soil diseases and soil profile associated with rhizosphere of rice (*Oryza sativa* Subsp. *Japonica*) growing paddy fields in Kansai region, Japan. Acta Agric. Scand. Sect. B Soil Plant Sci. 70, 444–455. doi: 10.1080/09064710.2020.1767192

[ref13] Ding S. Zhou D. Wei H. Wu S. Xie B. (2021). Alleviating soil degradation caused by watermelon continuous cropping obstacle: application of urban waste compost. Chemosphere 262:128387. doi: 10.1016/j.chemosphere.2020.128387, PMID: 33182114

[ref14] Dong L. Xu J. Feng G. Li X. Chen S. (2016). Soil bacterial and fungal community dynamics in relation to Panax notoginseng death rate in a continuous cropping system. Sci. Rep. 6:31802. doi: 10.1038/srep31802, PMID: 27549984 PMC4994099

[ref15] Du L. Huang B. Du N. Guo S. Shu S. Sun J. (2017). Effects of garlic/cucumber relay intercropping on soil enzyme activities and the microbial environment in continuous cropping. HortScience 52, 78–84. doi: 10.21273/HORTSCI11442-16

[ref16] Du J. X. Li Y. Ur-Rehman S. Mukhtar I. Yin Z. Dong H.. (2021). Synergistically promoting plant health by harnessing synthetic microbial communities and prebiotics. iScience 24:102918. doi: 10.1016/j.isci.2021.102918, PMID: 34430808 PMC8365361

[ref17] Du J. Li Y. Yin Z. Wang H. Zhang X. Ding X. (2020). High-throughput customization of plant microbiomes for sustainable agriculture. Front. Plant Sci. 11:569742. doi: 10.3389/fpls.2020.569742, PMID: 33013992 PMC7505944

[ref18] Dubey R. K. Dubey P. K. Abhilash P. C. (2019). Sustainable soil amendments for improving the soil quality, yield and nutrient content of *Brassica juncea* (L.) grown in different agroecological zones of eastern Uttar Pradesh, India. Soil Tillage Res. 195:104418. doi: 10.1016/j.still.2019.104418

[ref19] Dubey R. K. Dubey P. K. Chaurasia R. Rao C. S. Abhilash P. C. (2021). Impact of integrated agronomic practices on soil fertility and respiration on the Indo-Gangetic Plain of North India. Agronomy 11:402. doi: 10.3390/agronomy11020402

[ref20] Dubey R. K. Dubey P. K. Chaurasia R. Singh H. B. Abhilash P. C. (2020). Sustainable agronomic practices for enhancing the soil quality and yield of *Cicer arietinum* L. under diverse agroecosystems. J. Environ. Manag. 262:110284. doi: 10.1016/j.jenvman.2020.110284, PMID: 32250780

[ref21] Elmer W. H. Pignatello J. J. (2011). Effect of biochar amendments on mycorrhizal associations and Fusarium crown and root rot of asparagus in replant soils. Plant Dis. 95, 960–966. doi: 10.1094/PDIS-10-10-0741, PMID: 30732119

[ref22] Emmett B. Nelson E. B. Kessler A. Bauerle T. L. (2014). Fine-root system development and susceptibility to pathogen colonization. Planta 239, 325–340. doi: 10.1007/s00425-013-1989-7, PMID: 24170338

[ref23] Fan Z. (2019). Effects of Different Regulatory Measures on Tobacco Growth and Rhizosphere Soil Microflora. Zhengzhou: Zhengzhou University.

[ref24] Fang W. Song Z. Tao S. Zhang D. Huang B. Ren L.. (2020). Biochar mitigates the negative effect of chloropicrin fumigation on beneficial soil microorganisms. Sci. Total Environ. 738:139880. doi: 10.1016/j.scitotenv.2020.139880, PMID: 32531602

[ref25] Friedman J. (2017). Allelopathy, Autotoxicity, and Germination. London: Routledge, 629–644.

[ref26] Furtak K. Gajda A. M. (2018). Activity and variety of soil microorganisms depending on the diversity of the soil tillage system. Sustainability Agroecosyst. 45, 45–61. doi: 10.5772/intechopen.72966

[ref27] Galazka A. Gawryjolek K. Perzynski A. Galazka R. Ksiezak J. (2017). Changes in enzymatic activities and microbial communities in soil under long-term maize monoculture and crop rotation. Pol. J. Environ. Stud. 26, 39–46. doi: 10.15244/pjoes/64745

[ref28] Gao L. Liu X.-M. Du Y.-M. Zong H. Shen G.-M. (2019). Effects of tobacco–peanut relay intercropping on soil bacteria community structure. Ann. Microbiol. 69, 1531–1536. doi: 10.1007/s13213-019-01537-9

[ref29] Gao M. Yan Y. Li N. Luo P. Yang J. (2017). Effects of different tillage systems and amendments on root properties. IOP Conf. Seri. Mater. Sci. Eng. 207:012069. doi: 10.1088/1757-899X/207/1/012069

[ref30] Goring C. A. I. (2019). Chemical Control of Plant Pathogenic Microorganisms in Soil. Toronto: University of Toronto Press, 257–266.

[ref31] Griffin A. S. West S. A. Buckling A. (2004). Cooperation and competition in pathogenic bacteria. Nature 430, 1024–1027. doi: 10.1038/nature02744 15329720

[ref32] Gu S. Wei Z. Shao Z. Friman V. P. Cao K. Yang T.. (2020). Competition for iron drives phytopathogen control by natural rhizosphere microbiomes. Nat. Microbiol. 5, 1002–1010. doi: 10.1038/s41564-020-0719-8, PMID: 32393858 PMC7116525

[ref33] Han Q. H. Xin S. L. Hong H. (2010). Characterization of an antimicrobial material from a newly isolated bacillus amyloliquefaciens from mangrove for biocontrol of capsicum bacterial wilt. Biol. Control 54, 359–365. doi: 10.1016/j.biocontrol.2010.06.015

[ref34] He D. M. Chen Y. Yang S. P. Zhang X. Zhao J. Mo J. J.. (2017). Antifungal effects of three medicinal crops on Phytophthora nicotianae. Zhongguo Zhong Yao Za Zhi 42, 3509–3515. doi: 10.19540/j.cnki.cjcmm.20170814.010, PMID: 29218935

[ref35] He L. N. Liang Y. L. Gao J. Xiong Y. M. Zhou M. J. Wei Z. X. (2008). The effect of continuous cropping on yield, quality of cucumber and soil enzymes activities in solar greenhouse. J. Northwest A&F Univ. 36, 155–159.

[ref36] He H. Zhang S. Y. Shen W. Q. Zhu W. Noor I. Liu J. W.. (2021). Benzoic acid plays a part in rhizosphere microbial composition of peach seedlings grown in replanted soil. Rhizosphere 19:100364. doi: 10.1016/j.rhisph.2021.100364

[ref37] Hisashi K.-N. Keisuke N. Osamu O. Kiyotake S. Nobuyuki O. (2017). Asparagus decline: autotoxicity and autotoxic compounds in asparagus rhizomes. J. Plant Physiol. 213, 23–29. doi: 10.1016/j.jplph.2017.02.011 28314158

[ref38] Huang Y. Q. Han L. S. Han M. Xiao Y. N. Yang J. F. Han X. R. (2012). Influence of continuous cropping years on soil enzyme activities of peanuts. Chin. J. Oil Crop Sci. 34, 96–100.

[ref39] Hvězdová M. Kosubová P. Košíková M. Scherr K. E. Šimek Z. Brodský L.. (2018). Currently and recently used pesticides in central European arable soils. Sci. Total Environ. 613-614, 361–370. doi: 10.1016/j.scitotenv.2017.09.049 28917175

[ref40] Jangir C. Kumar S. Meena R. S. (2019). “Significance of soil organic matter to soil quality and evaluation of sustainability,” in Sustainable Agriculture. ed. Meena R. S. (Jodhpur, India: Scientific Publishers), 357–381.

[ref41] Jia H. T. Chen S. C. Yang S. Y. Shen Y. H. Qiao P. L. Wu F. Z.. (2018). Effects of vanillin on cucumber rhizosphere bacterial community. Allelopath. J. 44, 191–200. doi: 10.26651/allelo.j./2018-44-2-1164

[ref42] Jin H. (2010). Characterization of microbial life colonizing biochar and biochar-amended soils. Available at: https://hdl.handle.net/1813/17077

[ref43] Johannes L. Deborah A. B. Ingrid K. -K. Matthias C. R. (2020). The concept and future prospects of soil health. Nat. Rev. Earth Environ. 1, 544–553. doi: 10.1038/s43017-020-0080-8 33015639 PMC7116140

[ref44] Joseph E. C. Christopher A. W. Jennifer S. H. William T. P. Colin A. Edward R. B. (2018). Interactions among plants, bacteria, and fungi reduce extracellular enzyme activities under long-term N fertilization. Glob. Chang. Biol. 24, 2721–2734. doi: 10.1111/gcb.14081 29488286 PMC5980773

[ref45] Judith N. Donald L. S. (2020). Relevance of plant growth promoting microorganisms and their derived compounds, in the face of climate change. Agronomy 10:1179. doi: 10.3390/agronomy10081179

[ref46] Kicinska A. Wikar J. (2021). The effect of fertilizing soils degraded by the metallurgical industry on the content of elements in *Lactuca sativa* L. Sci. Rep. 11:4072. doi: 10.1038/s41598-021-83600-7, PMID: 33603123 PMC7893007

[ref47] Kumar S. Jakhar S. R. Dahiya S. Jangir C. K. Meena R. S. (2017). Soil sickness and productivity from ecological aspects. J. Pharma Phytochem. SPI 6, 827–831.

[ref48] Larkin R. P. (2008). Relative effects of biological amendments and crop rotations on soil microbial communities and soilborne diseases of potato. Soil Biol. Biochem. 40, 1341–1351. doi: 10.1016/j.soilbio.2007.03.005

[ref49] Li Z. Bai X. Jiao S. Li Y. Li P. Yang Y.. (2021). A simplified synthetic community rescues *Astragalus mongholicus* from root rot disease by activating plant-induced systemic resistance. Microbiome 9:217. doi: 10.1186/s40168-021-01169-9, PMID: 34732249 PMC8567675

[ref50] Li Y. Fang F. Wei J. Wu X. Cui R. Li G.. (2019). Humic acid fertilizer improved soil properties and soil microbial diversity of continuous cropping peanut: a three-year experiment. Sci. Rep. 9:12014. doi: 10.1038/s41598-019-48620-4, PMID: 31427666 PMC6700118

[ref51] Li W. Liu Q. (2019). Changes in fungal community and diversity in strawberry rhizosphere soil after 12 years in the greenhouse. J. Integr. Agric. 18, 677–687. doi: 10.1016/S2095-3119(18)62003-9

[ref52] Li X. Yang Z. Zhang Y. N. Yu L. Ding C. Liao Y.. (2020). Atractylodes lancea volatiles induce physiological responses in neighboring peanut plant during intercropping. Plant Soil 453, 409–422. doi: 10.1007/s11104-020-04615-z

[ref53] Liu Y. Jiang Y. Wang G. Zhang Y. Yang Y. Yue M.. (2016). Effect of different continuous cropping years on tobacco-growing soil’s physical and chemical properties and microflora. Chin. Agric. Sci. Bull. 32, 136–140. doi: 10.11924/j.issn.1000-6850.casb15110015

[ref54] Liu H. Li J. Carvalhais L. C. Percy C. D. Prakash Verma J. Schenk P. M.. (2021a). Evidence for the plant recruitment of beneficial microbes to suppress soil-borne pathogens. New Phytol. 229, 2873–2885. doi: 10.1111/nph.17057, PMID: 33131088

[ref55] Liu Z. Liu J. Yu Z. Yao Q. Li Y. Liang A.. (2020c). Long-term continuous cropping of soybean is comparable to crop rotation in mediating microbial abundance, diversity and community composition. Soil Tillage Res. 197:104503. doi: 10.1016/j.still.2019.104503

[ref56] Liu K. McInroy J. A. Hu C. H. Kloepper J. W. (2018). Mixtures of plant-growth-promoting Rhizobacteria enhance biological control of multiple plant diseases and plant-growth promotion in the presence of pathogens. Plant Dis. 102, 67–72. doi: 10.1094/PDIS-04-17-0478-RE, PMID: 30673446

[ref57] Liu H. Pan F. Han X. Song F. Zhang Z. Yan J.. (2020a). A comprehensive analysis of the response of the fungal community structure to long-term continuous cropping in three typical upland crops. J. Integr. Agric. 19, 866–880. doi: 10.1016/S2095-3119(19)62630-4

[ref58] Liu N. Shao C. Sun H. Liu Z. Guan Y. Wu L.. (2020b). Arbuscular mycorrhizal fungi biofertilizer improves American ginseng (*Panax quinquefolius* L.) growth under the continuous cropping regime. Geoderma 363:114155. doi: 10.1016/j.geoderma.2019.114155

[ref59] Liu S. Shi H. Wang L. Jiang T. Qi Y. (2019). Field control of bacterial strains LSN02 and LLGJ04 against tobacco soil-borne diseases. Mod. Agric. Sci. Technol. 75–76.

[ref60] Liu L. Sun C. Liu S. Chai R. Huang W. Liu X.. (2015a). Bioorganic fertilizer enhances soil suppressive capacity against bacterial wilt of tomato. PLoS One 10:e0121304. doi: 10.1371/journal.pone.0121304, PMID: 25830639 PMC4382293

[ref61] Liu S. Wang Z. Niu J. Dang K. Zhang S. Wang S.. (2021b). Changes in physicochemical properties, enzymatic activities, and the microbial community of soil significantly influence the continuous cropping of *Panax quinquefolius* L. (American ginseng). Plant Soil, 463, 427–446. doi: 10.1007/s11104-021-04911-2

[ref62] Liu W. X. Wang Q. L. Wang B. Z. Wang X. B. Franks A. E. Teng Y.. (2015b). Changes in the abundance and structure of bacterial communities under long-term fertilization treatments in a peanut monocropping system. Plant Soil 395, 415–427. doi: 10.1007/s11104-015-2569-3

[ref63] Ma W. Peng D. Walker S. L. Cao B. Gao C. H. Huang Q.. (2017). Bacillus subtilis biofilm development in the presence of soil clay minerals and iron oxides. NPJ Biofilms Microbiomes 3:4. doi: 10.1038/s41522-017-0013-6, PMID: 28649405 PMC5445608

[ref64] Ma Z. T. Wang Q. Wang X. W. Chen X. S. Wang Y. F. Mao Z. Q. (2021). Effects of biochar on replant disease by amendment soil environment. Commun. Soil Sci. Plant Anal. 52, 673–685. doi: 10.1080/00103624.2020.1869758

[ref65] Ma L. Zhang H. Zhou X. Yang C. Zheng S. Duo J.. (2018). Biological control tobacco bacterial wilt and black shank and root colonization by bio-organic fertilizer containing bacterium Pseudomonas aeruginosa NXHG29. Appl. Soil Ecol. 129, 136–144. doi: 10.1016/j.apsoil.2018.05.011

[ref66] Macik M. Gryta A. Frac M. (2020). “Biofertilizers in agriculture: an overview on concepts, strategies and effects on soil microorganisms,” in Advances in Agronomy. ed. Sparks D. L. (Elsevier), 31–87.

[ref67] Maier S. Tamm A. Wu D. Caesar J. Grube M. Weber B. (2018). Photoautotrophic organisms control microbial abundance, diversity, and physiology in different types of biological soil crusts. ISME J. 12, 1032–1046. doi: 10.1038/s41396-018-0062-8, PMID: 29445133 PMC5864206

[ref68] Manici L. M. Caputo F. Sacca M. L. (2017). Secondary metabolites released into the rhizosphere by Fusarium oxysporum and Fusarium spp. as underestimated component of nonspecific replant disease. Plant Soil 415, 85–98. doi: 10.1007/s11104-016-3152-2

[ref69] Mao N. Xue Q. Tang M. (2010). Biodegradation of benzoic acid and p-hydroxybenzoic acid in the strawberry planting soil by two strains of actinomyces. J. Northwest Agric. For. Univ. 38, 143–148.

[ref70] Mazzola M. Manici L. M. (2012). Apple replant disease: role of microbial ecology in cause and control. Annu. Rev. Phytopathol. 50, 45–65. doi: 10.1146/annurev-phyto-081211-173005, PMID: 22559069

[ref71] Michalojc Z. Buczkowska H. (2009). Content of macroelements in eggplant fruits depending on varied potassium fertilization. J. Elem. 14, 111–118. doi: 10.5601/jelem.2009.14.1.12

[ref72] Mikkel A. G. Jan K. V. Jeanette E. L. Waleed A. A.-S. Søren J. S. Peter S. (2015). Microbial diversity in a permanently cold and alkaline environment in Greenland. PLoS One 10:e0124863. doi: 10.1371/journal.pone.0124863 25915866 PMC4411134

[ref73] Miransari M. (2013). Soil microbes and the availability of soil nutrients. Acta Physiol. Plant. 35, 3075–3084. doi: 10.1007/s11738-013-1338-2

[ref74] Ouda S. Zohry A. Noreldin T. (2018). “Crop rotation maintains soil sustainability,” in Crop Rotation (Berlin: Springer), 55–76.

[ref75] Palansooriya K. N. Shaheen S. M. Chen S. S. Tsang D. C. W. Hashimoto Y. Hou D.. (2020). Soil amendments for immobilization of potentially toxic elements in contaminated soils: a critical review. Environ. Int. 134:105046. doi: 10.1016/j.envint.2019.105046, PMID: 31731004

[ref76] Pan Y. Cassman N. de Hollander M. Mendes L. W. Korevaar H. Geerts R. H.. (2014). Impact of long-term N, P, K, and NPK fertilization on the composition and potential functions of the bacterial community in grassland soil. FEMS Microbiol. Ecol. 90, 195–205. doi: 10.1111/1574-6941.12384, PMID: 25046442

[ref77] Pascale A. Proietti S. Pantelides I. S. Stringlis I. A. (2019). Modulation of the root microbiome by plant molecules: the basis for targeted disease suppression and plant growth promotion. Front. Plant Sci. 10:1741. doi: 10.3389/fpls.2019.01741, PMID: 32038698 PMC6992662

[ref78] Pervaiz Z. H. Iqbal J. Zhang Q. Chen D. Wei H. Saleem M. (2020). Continuous cropping alters multiple biotic and abiotic indicators of soil health. Soil Syst. 4:59. doi: 10.3390/soilsystems4040059

[ref79] Rasit A. Kenya K. Solomon S. O. Bahar S. Jun M. Kazuki S.. (2021). Top-down effects of protists are greater than bottom-up effects of fertilisers on the formation of bacterial communities in a paddy field soil. Soil Biol. Biochem. 156:108186. doi: 10.1016/j.soilbio.2021.108186

[ref80] Ren D. Madsen J. S. Sørensen S. J. Burmølle M. (2015). High prevalence of biofilm synergy among bacterial soil isolates in cocultures indicates bacterial interspecific cooperation. ISME J. 9, 81–89. doi: 10.1038/ismej.2014.96, PMID: 24936766 PMC4274433

[ref81] Rial C. Novaes P. Varela R. M. Molinillo J. M. Macias F. A. (2014). Phytotoxicity of cardoon (Cynara cardunculus) allelochemicals on standard target species and weeds. J. Agric. Food Chem. 62, 6699–6706. doi: 10.1021/jf501976h, PMID: 24974850

[ref82] Sadikshya D. Gao S. Duan Y. Wang D. (2020). Soil microbial community structure affected by biochar and fertilizer sources. Appl. Soil Ecol. 150:103452. doi: 10.1016/j.apsoil.2019.103452

[ref83] Sassone-Corsi M. Raffatellu M. (2015). No vacancy: how beneficial microbes cooperate with immunity to provide colonization resistance to pathogens. J. Immunol. 194, 4081–4087. doi: 10.4049/jimmunol.1403169, PMID: 25888704 PMC4402713

[ref84] Schippers B. A. Bakker A. W. Bakker P. (1987). Interactions of deleterious and beneficial Rhizosphere microorganisms and the effect of cropping practices. Annu. Rev. Phytopathol. 25, 339–358. doi: 10.1146/annurev.py.25.090187.002011

[ref85] Serpil S. (2012). Investigation of effect of chemical fertilizers on environment. APCBEE Proc. 1, 287–292. doi: 10.1016/j.apcbee.2012.03.047

[ref86] Shen W. S. Ni Y. Y. Gao N. Bian B. Y. Zheng S. A. Lin X. G.. (2016). Bacterial community composition is shaped by soil secondary salinization and acidification brought on by high nitrogen fertilization rates. Appl. Soil Ecol. 108, 76–83. doi: 10.1016/j.apsoil.2016.08.005

[ref87] Shen G. Zhang S. Liu X. Jiang Q. Ding W. (2018). Soil acidification amendments change the rhizosphere bacterial community of tobacco in a bacterial wilt affected field. Appl. Microbiol. Biotechnol. 102, 9781–9791. doi: 10.1007/s00253-018-9347-0, PMID: 30302520 PMC6208964

[ref88] Shen L. Zhu G. Guo S. Li X. Xiao S. Xu J.. (2020). Isolation of a Pseudomonas putida strain that degrades p-hydroxybenzoic acid from the soil of a Panax ginseng field. [Preprint].

[ref89] Stéphane C. Günter B. Saima M. Angela S. Ahmed L. Florence M. (2013). Use of beneficial bacteria and their secondary metabolites to control grapevine pathogen diseases. BioControl 58, 435–455. doi: 10.1007/s10526-012-9479-6

[ref90] Syrie M. H. Hannah L. B. Bradley S. C. Fiona C.-C. Matthew T. Gavin L. (2017). Bacteria as emerging indicators of soil condition. Appl. Environ. Microbiol. 83:e02826-16. doi: 10.1128/AEM.02826-16 27793827 PMC5165110

[ref91] Tan Y. Cui Y. Li H. Kuang A. Li X. Wei Y.. (2017). Rhizospheric soil and root endogenous fungal diversity and composition in response to continuous Panax notoginseng cropping practices. Microbiol. Res. 194, 10–19. doi: 10.1016/j.micres.2016.09.009, PMID: 27938858

[ref92] Venkatesh C. Pradeep V. (2016). Microbial fuel cell: a green approach for the utilization of waste for the generation of bioelectricity. Bioresour. Bioprocess. 3:38. doi: 10.1186/s40643-016-0116-6

[ref93] Victoria H. W. Fiona K. B. Matthew J. S. Sarah D. A. Huiya G. Brian W. V.. (2013). Biocommodities from photosynthetic microorganisms. Environ. Prog. Sustain. Energy 32:11849. doi: 10.1002/ep.11849

[ref94] Wang H. Chen Y. Wang S. Li C. Sun X. Li S. (2010). Control effects of chloropicrin soil fumigation on tobacco weeds and soil-borne diseases. Chin. Agric. Sci. Bull. 26, 244–248.

[ref95] Wang M. Jiang C. Pan W. (2008). Studing on physico-chemical properties and microbiological community in tobacco-growing soils under different continuous cropping years. J. Anhui. Agric. Sci. 12, 5033–5034. doi: 10.3724/SP.J.1005.2008.01083

[ref96] Wang H. H. Ren T. B. Yang H. J. Feng Y. Q. Feng H. L. Liu G. S.. (2020a). Research and application of biochar in soil CO_2_ emission, fertility, and microorganisms: a sustainable solution to solve China’s agricultural straw burning problem. Sustainability 12:1922. doi: 10.3390/su12051922

[ref97] Wang W. Wang Z. Yang K. Wang P. Wang H. Guo L.. (2020b). Biochar application alleviated negative plant-soil feedback by modifying soil microbiome. Front. Microbiol. 11:799. doi: 10.3389/fmicb.2020.00799, PMID: 32411119 PMC7201025

[ref98] Wang M. Y. Wu C. N. Cheng Z. H. Meng H. W. (2015). Growth and physiological changes in continuously cropped eggplant (*Solanum melongena* L.) upon relay intercropping with garlic (*Allium sativum* L.). Front. Plant Sci. 6:262. doi: 10.3389/fpls.2015.00262, PMID: 25964788 PMC4408842

[ref99] Wang R. Q. Xiao Y. P. Lv F. J. Hu L. Y. Wei L. G. Yuan Z. Q.. (2018b). Bacterial community structure and functional potential of rhizosphere soils as influenced by nitrogen addition and bacterial wilt disease under continuous sesame cropping. Appl. Soil Ecol. 125, 117–127. doi: 10.1016/j.apsoil.2017.12.014

[ref100] Wang Y. Zhang Y. Li Z. Zhao Q. Huang X. Huang K. (2020c). Effect of continuous cropping on the rhizosphere soil and growth of common buckwheat. Plant Prod. Sci. 23, 81–90. doi: 10.1080/1343943X.2019.1685895

[ref101] Wang J. Zhang Z. Liu Y. (2018a). Spatial shifts in grain production increases in China and implications for food security. Land Use Policy 74, 204–213. doi: 10.1016/j.landusepol.2017.11.037

[ref102] Wang R. Zhang H. Sun L. Qi G. Chen S. Zhao X. (2017). Microbial community composition is related to soil biological and chemical properties and bacterial wilt outbreak. Sci. Rep. 7, 1–10. doi: 10.1038/s41598-017-00472-6 28336973 PMC5428506

[ref103] Wang Y. Zhang W. Zhang Z. Wang W. Xu S. He X. (2021). Isolation, identification and characterization of phenolic acid-degrading bacteria from soil. J. Appl. Microbiol. 131, 208–220. doi: 10.1111/jam.14956, PMID: 33270328

[ref104] Weiβ S. Bartsch M. Winkelmann T. (2017). Transcriptomic analysis of molecular responses in Malus domestica ‘M26’ roots affected by apple replant disease. Plant Mol. Biol. 94, 303–318. doi: 10.1007/s11103-017-0608-6 28424966

[ref105] Wu W. (2010). Research on Tobacco Autotoxic Substances and Their Effects on Rhizosphere Soil Microorganisms. Fuzhou: Fujian Agriculture and Forestry University, M1 - Master’s degree.

[ref106] Wu Y. Cai P. Jing X. Niu X. Ji D. Ashry N. M.. (2019). Soil biofilm formation enhances microbial community diversity and metabolic activity. Environ. Int. 132:105116. doi: 10.1016/j.envint.2019.105116, PMID: 31479959

[ref107] Wu Z. J. Xie Z. K. Yang L. Wang R. Y. Guo Z. H. Zhang Y. B.. (2015). Identification of autotoxins from root exudates of Lanzhou lily (Lilium davidii var. unicolor). Allelopath. J. 35, 35–48.

[ref108] Xi H. Shen J. Qu Z. Yang D. Liu S. Nie X.. (2019). Effects of long-term cotton continuous cropping on soil microbiome. Sci. Rep. 9:18297. doi: 10.1038/s41598-019-54771-1, PMID: 31797982 PMC6892916

[ref109] Xia J. Ni C. Liu S. (2019). Research progress on application effect of biomass charcoal and its restoration of soil phenolic acid pollution. Plant Dis. Pests 10, 5–9. doi: 10.19579/j.cnki.plant-d.p.2019.01.002

[ref110] Xie X. Dai C. (2015). Biodegradation of a model allelochemical cinnamic acid by a novel endophytic fungus Phomopsis liquidambari. Int. Biodeterior. Biodegradation 104, 498–507. doi: 10.1016/j.ibiod.2015.08.004

[ref111] Xie H. Ku Y. Yang X. Cao L. Mei X. Yang N.. (2020). Alleviating Continuous Monocropping Obstacle in Melon: Biological Elimination of Phenolic Acid. [Preprint].

[ref112] Yan Z. Wang H. He B. Liu M. Wang G. Chen X.. (2012). Study on controlling measurement for continuous cropping obstacle in Traditional Chinese Medicinal plants by micro-ecological research model. Pharm. Clin. Chin. Mater. Med. 2.

[ref113] Yang C. Dong Y. Friman V. P. Jousset A. Wei Z. Xu Y.. (2019). Carbon resource richness shapes bacterial competitive interactions by alleviating growth-antibiosis trade-off. Funct. Ecol. 33, 868–875. doi: 10.1111/1365-2435.13292

[ref114] Yim B. Smalla K. Winkelmann T. (2013). Evaluation of apple replant problems based on different soil disinfection treatments-links to soil microbial community structure? Plant Soil 366, 617–631. doi: 10.1007/s11104-012-1454-6

[ref115] Yin H. Yi Y. Luo K. Kuang C. Deng Z. (2004). Identification and biocontrol test of tobacco endophytic bacteria against *Ralstonia solanacearum*. Chin. J. Biol. Control 20, 219–220.

[ref116] Yu Y. Yang J. Zeng S. Wu D. Douglass F. J. Joshua L. S. (2017). Soil pH, organic matter, and nutrient content change with the continuous cropping of Cunninghamia lanceolata plantations in South China. J. Soils Sediments 17, 2230–2238. doi: 10.1007/s11368-016-1472-8

[ref117] Zeng J. Liu J. Lu C. Ou X. Luo K. Li C.. (2020). Intercropping with turmeric or ginger reduce the continuous cropping obstacles that affect Pogostemon cablin (patchouli). Front. Microbiol. 11:579719. doi: 10.3389/fmicb.2020.579719 33133047 PMC7578394

[ref118] Zhang D. (2015). “Analysis on the Influencing Factors of Farmers' Income in Heilongjiang.” in *2nd International Conference on Civil, Materials and Environmental Sciences*. March 13-14, 2015.

[ref119] Zhang Q. Gao X. Ren Y. Ding X. Qiu J. Li N.. (2018). Improvement of *Verticillium* wilt resistance by applying arbuscular mycorrhizal fungi to a cotton variety with high symbiotic efficiency under field conditions. Int. J. Mol. Sci. 19:241. doi: 10.3390/ijms19010241, PMID: 29342876 PMC5796189

[ref120] Zhang S. Jiang Q. Liu X. Liu L. Ding W. (2020). Plant growth promoting rhizobacteria alleviate aluminum toxicity and ginger bacterial wilt in acidic continuous cropping soil. Front. Microbiol. 11:569512. doi: 10.3389/fmicb.2020.569512, PMID: 33424780 PMC7793916

[ref121] Zhang B. Li X. Wang F. Li M. Zhang J. Gu L.. (2016). Assaying the potential autotoxins and microbial community associated with Rehmannia glutinosa replant problems based on its ‘autotoxic circle’. Plant Soil 407, 307–322. doi: 10.1007/s11104-016-2885-2

[ref122] Zhang X. L. Pan Z. G. Zhou X. F. Ni W. (2007). Autotoxicity and continuous cropping obstacles: a review. Chin. J. Soil Sci. 4, 781–784. doi: 10.3321/j.issn:0564-3945.2007.04.033

[ref123] Zhang Q.-C. Shamsi I. H. Xu D.-T. Wang G.-H. Lin X.-Y. Jilani G.. (2012). Chemical fertilizer and organic manure inputs in soil exhibit a vice versa pattern of microbial community structure. Appl. Soil Ecol. 57, 1–8. doi: 10.1016/j.apsoil.2012.02.012

[ref124] Zhang H. Wang R. Chen S. Qi G. He Z. Zhao X. (2017). Microbial taxa and functional genes shift in degraded soil with bacterial wilt. Sci. Rep. 7:39911. doi: 10.1038/srep39911, PMID: 28051173 PMC5209727

[ref125] Zhang Y. Zheng Y. Xia P. Xun L. Liang Z. (2019). Impact of continuous Panax notoginseng plantation on soil microbial and biochemical properties. Sci. Rep. 9:13205. doi: 10.1038/s41598-019-49625-9, PMID: 31519939 PMC6744506

[ref126] Zhao J. Ni T. Li Y. Xiong W. Ran W. Shen B.. (2014). Responses of bacterial communities in arable soils in a rice-wheat cropping system to different fertilizer regimes and sampling times. PLoS One 9:e85301. doi: 10.1371/journal.pone.0085301, PMID: 24465530 PMC3896389

[ref127] Zhao Y. Wu L. Chu L. Yang Y. Li Z. Azeem S.. (2015). Interaction of Pseudostellaria heterophylla with Fusarium oxysporum f.sp. heterophylla mediated by its root exudates in a consecutive monoculture system. Sci. Rep. 5:8197. doi: 10.1038/srep08197, PMID: 25645742 PMC4314652

[ref128] Zhenli L. H. Xiaoe E. Y. Peter J. S. (2005). Trace elements in agroecosystems and impacts on the environment. J. Trace Elem. Med. Biol. 19, 125–140. doi: 10.1016/j.jtemb.2005.02.010 16325528

[ref129] Zhou X. Guo J. Wang M. Li J. Zhang H. Wang X. (2016). Study on the effects of biocontrol agent to control major soil-borne diseases of tobacco. Crop. Res. 30, 176–180. doi: 10.16848/j.cnki.issn.1001-5280.2016.02.17

[ref130] Zhou X. Wu F. (2012). Dynamics of the diversity of fungal and Fusarium communities during continuous cropping of cucumber in the greenhouse. FEMS Microbiol. Ecol. 80, 469–478. doi: 10.1111/j.1574-6941.2012.01312.x, PMID: 22273443

[ref131] Zhu Y. M. Fazio G. Mazzola M. (2014). Elucidating the molecular responses of apple rootstock resistant to ARD pathogens: challenges and opportunities for development of genomics-assisted breeding tools. Hort. Res. 1:14043. doi: 10.1038/hortres.2014.43, PMID: 26504547 PMC4596329

